# The potential role of sciatic nerve stiffness in the limitation of maximal ankle range of motion

**DOI:** 10.1038/s41598-018-32873-6

**Published:** 2018-09-28

**Authors:** Ricardo J. Andrade, Sandro R. Freitas, François Hug, Guillaume Le Sant, Lilian Lacourpaille, Raphäel Gross, Peter McNair, Antoine Nordez

**Affiliations:** 1 Laboratory ≪Movement, Interactions, Performance≫ (EA 4334), Faculty of Sport Sciences, University of Nantes, Nantes, France; 2 Universidade de Lisboa, Faculdade de Motricidade Humana, CIPER, P-1100 Lisbon, Portugal; 3 Institut Universitaire de France (IUF), Paris, France; 4 The University of Queensland, NHMRC Centre of Clinical Research Excellence in Spinal Pain, Injury and Health, School of Health and Rehabilitation Sciences, Brisbane, Australia; 5 School of Physiotherapy (IFM3R), Nantes, France; 6 Gait Analysis Laboratory, Physical and Rehabilitation Medicine Department, University Hospital of Nantes, Nantes, France; 7 Health and Rehabilitation Research Institute, Faculty of Health and Environmental Sciences, Auckland University of Technology, Auckland, New Zealand

## Abstract

It is a long held belief that maximal joint range of motion (ROM) is restricted by muscle tension. However, it exists indirect evidence suggesting that this assumption may not hold true for some joint configurations where non-muscular structures, such as the peripheral nerves, are stretched. Direct evidences are lacking. This study aimed to determine whether a static stretching aiming to load the sciatic nerve without stretch within plantar flexors is effective to: (i) alter nerve stiffness; and (ii) increase the ankle’s maximal ROM. Passive maximal ankle ROM in dorsiflexion was assessed with the hip flexed at 90° (HIP-flexed) or neutral (HIP-neutral, 0°). Sciatic nerve stiffness was estimated using shear wave elastography. Sciatic nerve stretching induced both a 13.3 ± 7.9% (P < 0.001) decrease in the nerve stiffness and a 6.4 ± 2.6° increase in the maximal dorsiflexion ROM assessed in HIP-flexed. In addition, the decrease in sciatic nerve stiffness was significantly correlated with the change in maximal ROM in dorsiflexion (r = −0.571, P = 0.026). These effects occurred in the absence of any change in *gastrocnemius medialis* and *biceps femoris* stiffness, and ankle passive torque. These results demonstrate that maximal dorsiflexion ROM can be acutely increased by stretching the sciatic nerve, without altering the muscle stiffness.

## Introduction

The maximal range of motion (ROM) of a joint is an important functional parameter to estimate the maximal muscle length or muscle extensibility that is widely used in clinical practice, sport training and research^1^. It is a long-held belief that maximal ankle ROM in dorsiflexion is restricted by the tension within the plantar flexor muscles, i.e. either the maximal tension that can be reached (i.e., “mechanical theory”) or the perception of this tension (i.e., “sensory theory”)^2^. However, although during ankle rotations many other non-muscular tissues (e.g., peripheral nerves and lower limb *fasciae*) experience large changes in their length and tension^3–6^, the influence of these non-muscular tissues on the maximal ROM at a joint remains largely unexplored.

Two previous studies observed a remarkable decrease in the maximal ankle ROM in dorsiflexion when the hip was flexed from the neutral position to 90°^7,8^. This change occurred in the absence of any changes in the *gastrocnemius medialis* (GM) local stiffness and ankle torque^8^. Because there is no muscle-tendon unit crossing both the hip and ankle joints, these results have suggested that non-muscular structures may play a role in the limitation of maximal ankle ROM in dorsiflexion^9^. A possible candidate to explain this effect is the sciatic nerve that extends from the spine through branches to the foot, and like most other connective tissues, exhibits visco-elastic behavior^10,11^. If so, a sciatic nerve stretch may decrease the stiffness of the neural tissue and improve the maximal ankle dorsiflexion ROM.

To test this hypothesis non-invasively, it is necessary to assess the sciatic nerve stiffness during passive ankle rotations. Ultrasound shear wave elastography can be used to assess *in vivo* the shear wave velocity within soft tissues^12,13^, this parameter being directly related to the shear modulus of the tissue^14^. It therefore provides an accurate characterization of muscle stiffness^15^. Recently, it was showed that this technique can be used to reliably measure the shear wave velocity within the sciatic nerve during passive ankle rotations^4^. A better understanding of the biomechanical properties of peripheral nerves *in vivo* would have strong clinical importance, in particular to the management of nerves in the context of entrapment neuropathy or chronic neuropathies where both nerve’s stiffness and maximal joint ROM might be affected^16–18^.

This study was designed to appreciate the role of the sciatic nerve stiffness in the maximal ankle ROM in dorsiflexion. An experimental protocol was conducted in humans to determine whether a total of 6-min static stretch that aims to load the sciatic nerve without stretch within plantar flexors is effective to accutely: (i) alter nerve stiffness; and (ii) increase the ankle’s maximal ROM. The sciatic stiffness was measured, before and after the 6-min static stretch, while the nerve was progressively stretched during ankle dorsiflexion. We hypothesized that: (i) it is possible to selectively decrease the sciatic nerve stiffness without altering the muscle stiffness; and (ii) the specific sciatic stretch would induce an increase in maximal ankle ROM in dorsiflexion.

## Results

**Figure 1:**
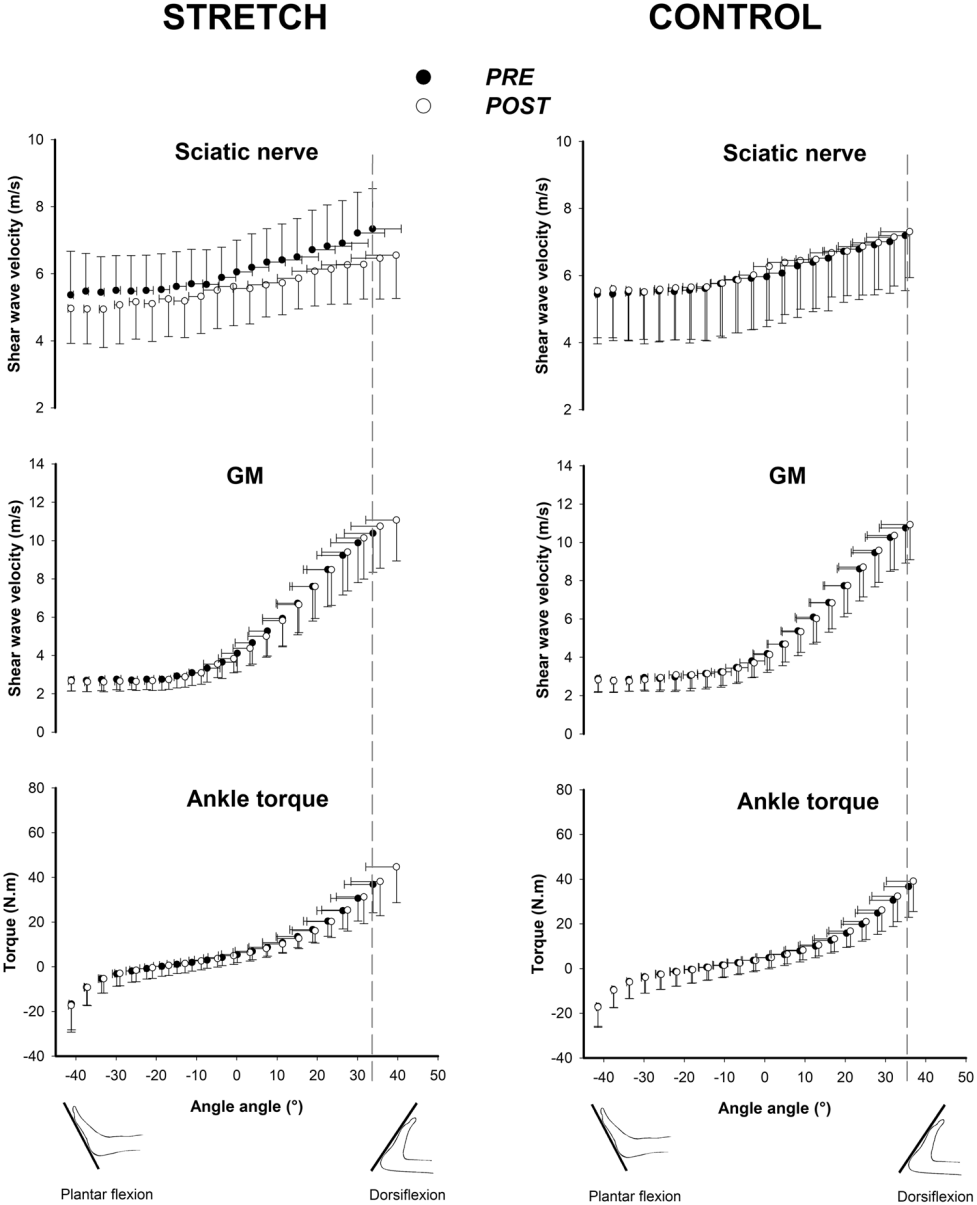
Relationships between the ankle angle and the sciatic nerve and gastrocnemius medialis (GM) shear wave velocity; and ankle torque. Measurements were performed before and immediately after the intervention in both Stretch and Control sessions. All assessments were performed in HIP-neutral position. The ankle rotation started at 40° of plantar flexion. The dotted gray line represents the ankle angle used to statistical analysis, corresponding to 100% of maximal ankle ROM in dorsiflexion reached in the pre-testing.

The effects of nerve stretching on ankle torque and sciatic nerve, GM and BF shear wave velocity are summarized in Table 1 and Fig. 1.

**Table 1:** Ankle passive torque, sciatic nerve, gastrocnemius medialis (GM) and biceps femoris long head (BF) shear wave velocity, and sciatic nerve thickness at the maximal ankle dorsiflexion ROM, before and after the Stretch and Control interventions.

	Ankle angle*	Torque (N.m)		Sciatic nerve SWV (m/s)^#^			GM SWV (m/s)		BF SWV (m/s)		Sciatic nerve thickness (cm)	
		Before	After	Before	After	P-value	Before	After	Before	After	Before	After
Stretch session	32.9 ± 7.3°	35.3 ± 12.7	33.4 ± 13.7	7.3 ± 0.3	6.3 ± 0.3	0.00001	18.1 ± 3.5	18.1 ± 3.6	5.1 ± 1.0	5.1 ± 0.8	0.5 ± 0.1	0.5 ± 0.1
Control session	34.0 ± 6.3°	34.8 ± 13.3	35.5 ± 13.5	7.3 ± 0.3	7.4 ± 0.4	0.486	19.1 ± 2.2	19.0 ± 2.2	5.4 ± 1.2	5.4 ± 1.1	0.5 ± 0.1	0.5 ± 0.1

Values are presented as mean + SD. *Ankle angles corresponded to 100% of pre-testing maximal ankle ROM in dorsiflexion. Session × time interaction was found only for the sciatic nerve SWV (^#^*p* < *0*.*0001*). *Post hoc* p-values for the before to after intervention changes in sciatic nerve shear-wave velocity (SWV) are presented for both Stretch and Control sessions. No significant session × time interactions were observed for ankle torque, GM and BF shear wave velocity, and sciatic nerve thickness. GM: *gastrocnemius medialis*; BF: *biceps femoris*; SWV: shear wave velocity; ROM: range of motion.

### Muscle shear wave velocity

There was neither a significant session × time interaction (GM: P = 0.660; BF: P = 0.855) nor a significant main effects of time (GM: P = 0.742; BF: P = 0.696) and session (GM: P = 0.317; BF: P = 0.2) on GM and BF shear wave velocity measured at 100% of the pre-testing ankle ROM in dorsiflexion (Table 1; Fig. 1).

### Sciatic nerve shear wave velocity

A significant session × time interaction was observed on the sciatic nerve shear wave velocity (P < 0.0001; _p_η^2^ = 0.658). *Post hoc* tests revealed that the sciatic nerve shear wave velocity was 13.3 ± 7.9% lower (*p* < 0.0001) after the nerve stretching, while no significant changes (P = 0.486) were observed in the Control session.

### Sciatic nerve nerve thickness

Neither significant session × time interactions (P = 1), nor main effects of session (P = 0.747) and time (P = 0.538) were observed for the sciatic nerve thickness. This result confirms that acute changes in shear wave velocity after stretch intervention were not influenced by the nerve thickness.

### Ankle torque

A significant session × time (P = 0.042; _p_η^2^ = 0.262) interaction was observed at 100% of the pre-testing ankle ROM in dorsiflexion. However, post hoc tests showed no significant effect of the intervention on the passive torque (P = 0.25 [Cohen’s *d* = 0.15] and P = 1 [Cohen’s *d* = 0.06] for the Stretch and Control sessions, respectively), confirming that it was not significantly affected.

### Maximal ankle ROM in dorsiflexion

**Figure 2:**
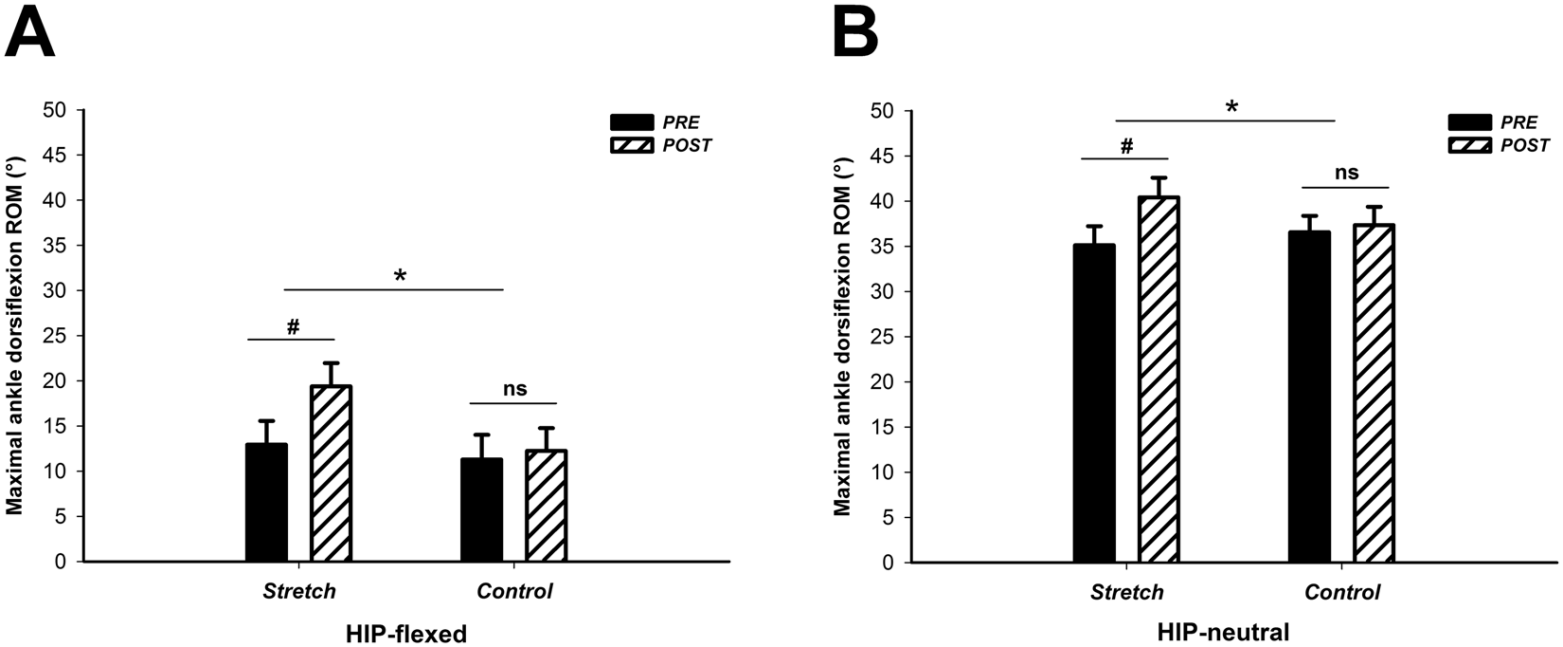
(**A**) For the HIP-flexed position, the observed session × time interaction (*p < 0.0001) demonstrates a significant increase in the maximal dorsiflexion range of motion (ROM) after the intervention performed in the Stretch session (^#^*p* < 0.0001); (**B**) For the HIP-neutral, the interaction session × time (*p = 0.010) shows that the maximum dorsiflexion ROM increases after the Stretch session (^#^*p* = 0.001).

A significant session × time interactions were observed on the maximal ankle ROM in dorsiflexion for both HIP-neutral and HIP-flexed positions [P = 0.010 (_p_η^2^ = 0.384) and P < 0.0001 (_p_η^2^ = 0.644), respectively]. For HIP-flexed, *post hoc* analysis revealed a significant increase (+6.4° ± 2.4°; *p* < 0.0001) in maximal ankle ROM in dorsiflexion immediately after the intervention performed during the Stretch session, but no significant changes were observed during the Control session (Fig. 2A). Additionally, p*ost hoc* tests showed that the maximal ankle ROM in dorsiflexion assessed in HIP-neutral position increased after the intervention performed in the Stretch session (+5.3° ± 4.2°; *p* = 0.001), but no differences were observed in Control session (Fig. 2B).

**Figure 3:**
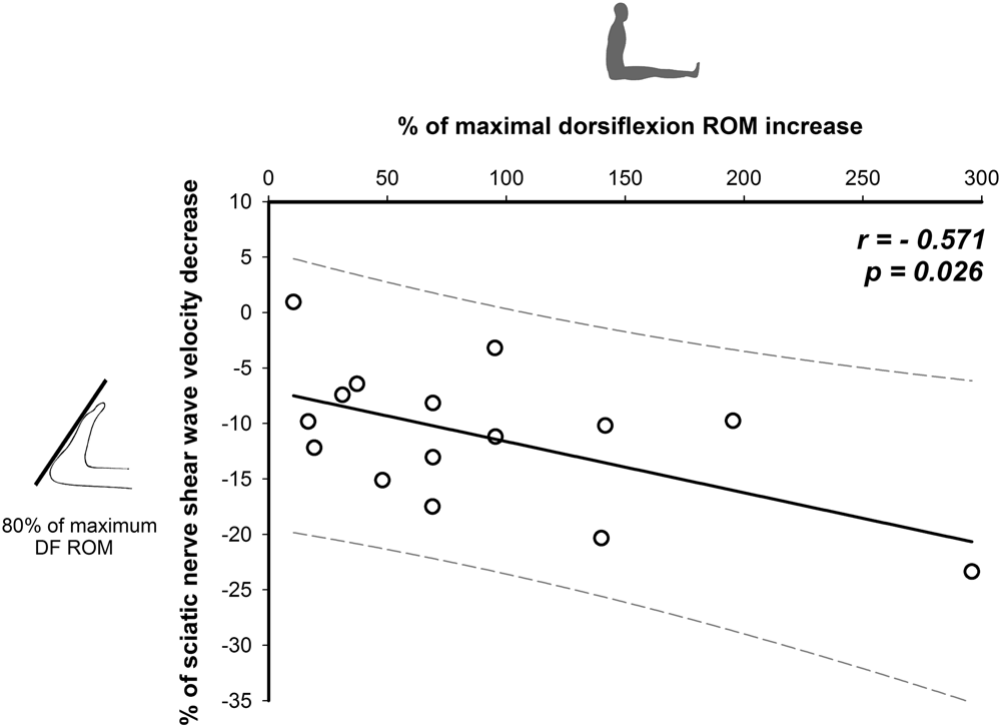
The effects of nerve stretching technique on local sciatic nerve stiffness and maximal ankle ROM in dorsiflexion. The negative correlation coefficient shows that the nerve stretching induced an increase (in %) in maximal ankle ROM in dorsiflexion measured in HIP-flexed position that was correlated with the percentage decrease in sciatic nerve stiffness (Stretch session).

A significant negative correlation (r = −0.571, P = 0.026) was observed between the relative decrease (in %) in sciatic nerve shear wave velocity observed after the nerve stretch (Stretch session) and the relative increase (in %) in maximal ROM in dorsiflexion assessed in HIP-flexed position (Fig. 3). In contrast, there was no significant correlation for the Control session (−0.248, P = 0.373). In addition, there was no significant correlation between the changes in shear wave velocity and the changes in maximal ROM assessed in HIP-neutral position in both Stretch (r = 0.072, P = 0.798) and Control (r = −0.038, P = 0.892) sessions.

### RMS EMG

Neither significant session × time interactions, nor main effects of session and time were observed for GL (1.17% ± 1.18% of maximal EMG amplitude), SOL (2.85% ± 2.78% of maximal EMG amplitude), ST (0.56% ± 0.58% of maximal EMG amplitude) nor TA (0.89% ± 1.35% of maximal EMG amplitude) at 100% of the pre-testing ankle ROM in dorsiflexion.

## Discussion

The sciatic nerve stretching performed in this study was designed to directly manipulate the sciatic stiffness, and to examine the immediate effects of decreased nerve stiffness on the maximal ankle ROM in dorsiflexion. In accordance with our hypothesis, we observed that it is possible to alter the maximal ankle ROM in dorsiflexion without affecting the passive stiffness of plantar flexors muscles. This study provides the first *in vivo* experimental evidence that stretching peripheral nerves is efficient to improve the maximal ROM at a joint.

In the present study, the sciatic nerve stretch was effective to notably increase the maximal ankle ROM in dorsiflexion in the HIP-flexed position (88.9% ± 77.8%), in which the sciatic nerve must be maximally stretched. Surprisingly, the maximal ROM also improved, but with lower magnitude (17% ± 15.1%), in HIP-neutral position (Fig. 2B), where the nerve load at the maximal ROM in dorsiflexion is considerably lower^8^. Although it is a long-held belief that the maximal ankle ROM in dorsiflexion assessed in HIP-neutral position is mainly explained by the tension developed by the plantar flexor muscles^2^, our findings demonstrate that the mechanical properties of non-muscular structures, such as peripheral nerves, may also contribute to the maximal ROM. However, the sciatic nerve stretch can modify not only the nerve’s mechanical behavior but also its sensory properties increasing the stretch tolerance, even in a HIP-neutral position. This is shown in Fig. 1 with the exponential increase in sciatic nerve stiffness observed during passive ankle dorsiflexion while the participants were positioned in HIP-neutral position. Using similar testing at the same HIP-neutral position, Andrade *et al*.^4^ showed that the steep increase in nerve stiffness occurs from ~50% of ankle’s maximum dorsiflexion ROM. These findings demonstrate that the sciatic nerve is also loaded during ankle dorsiflexion in HIP-neutral position. Therefore, the decrease in sciatic stiffness observed in our study immediately after the stretch intervention can have played a role in the maximal ROM assessed in the HIP-neutral position. Conversely, it has long been suggested that stretch tolerance is a potential mechanism for increasing maximal ROM following stretching^2^. Indeed, peripheral nerves sheaths are innervated by intrinsic nerves, such as the nervi nervorum, that are very sensitive to stretching in the long axis of the nerves^19,20^. Therefore, it is also possible that stretching of nerve sheaths have triggered the *nervi nervorum* and changed, acutely, its mechanosensitivity threshold to nerve tensioning. This is likely to induce changes in stretch amplitude for the subsequent loading cycle due to increased tolerance for a given instruction (e.g. onset of pain). Although the mechanisms underlying increased stretch tolerance are not fully understood, generalized desensitization affecting non-local tissues and/or involving cortical activity have been suggested to explain enhanced maximal ROM after acute stretching^21,22^.

We designed an experimental protocol to stretch the sciatic nerve without affecting plantar flexor muscles. It is accepted that lower limb position (i.e. hip, knee and ankle) may influence the loading of the sciatic nerve tract^3–5,23,24^. Based on these cadaver and ultrasound findings, the sciatic nerve tract was stretched by manipulating the position of the pelvis, trunk and cervical spine. In regard to the position of the ankle joint during testing, although previous elastography studies have demonstrated that plantar flexor muscles are minimally stretched when the ankle is in neutral position^25,26^, it is unlikely that the stretching magnitude was sufficient to modify the muscles’ mechanical properties^27^. This was confirmed by the fact that no significant changes of both ankle torque and muscle shear modulus were observed after the intervention in both the stretch and the control sessions; while the sciatic nerve stiffness decreased considerably immediately after the Stretch session. Taken together, these results support that the stretching technique implemented in the present study can target the sciatic nerve with non-detectable mechanical changes in plantar flexors and knee flexors muscles. To our knowledge, this study is the first that experimentally validated *in-vivo* a stretching protocol dedicated to non-muscular structures of the lower-limb, such as the peripheral nerves, acting on ankle flexibility.

The specific physiological explanations for a change in nerve stiffness after the Stretch session remain somewhat speculative. Various *in-vitro* and *in-situ* works showed that nerves exhibit a non-linear stress-strain behavior and time-dependent viscoelastic properties when stretched to a fixed strain^10,28–31^. These findings suggest that nerve possesses a similar viscoelastic behavior to the muscle which can explain an acute decrease in stiffness while being stretched. In accordance with our hypothesis, the decreased sciatic nerve stiffness was accompanied with an overall increase in ROM in dorsiflexion, notably in HIP-flexed position. As no changes occurred in plantar flexors muscles, we are confident that this change in maximal ROM in dorsiflexion is due to non-muscular structures. Of note, the posture used for elastography assessments induce very low tension on biceps femoris, which is demonstrated by the absence of change in shear wave velocity. Thus, it is not likely that changes in sciatic shear wave velocity were induced by surrounding biceps femoris passive tension. However, although we can conclude that the sciatic nerve stiffness plays an important role on maximal ROM, the cumulative effect of other non-muscular structures (not evaluated in this study) remains inconclusive. Interestingly, recent studies have shown that nerve stiffness is increased in pathologies where a joint’s maximal ROM is known to be decreased^16,17,32^. This suggests that adequate nerve stretching techniques may be a useful treatment modality in the management of certain neuropathies or in sports where large ROM is required.

Like peripheral nerves, fascial tissue is continuous from the trunk and extends throughout the lower limbs^33^. As such, they are likely to undergo mechanical deformation with specific body configurations. For instance, the displacement of the gastrocnemius medialis deep fascia has been shown to be highly correlated with pelvic motion (i.e. forward tilting) confirming *in-vivo* a myofascial connectivity between the trunk and lower leg^6^, as reported in anatomical studies^33^. In addition, Wilke *et al*. (2017) recently found that an acute bout of hamstring and plantar flexors stretches is as effective as local neck stretching to improve cervical maximal ROM. Although the authors did not assess the mechanical properties of the stretched tissues, their results can be attributed to the involvement of fasciae structures between the lower limb and the spine and are supported by anatomical findings^33^. Together, these results suggest that fascial tissues, which behave mechanically like other connective tissues^33^, are likely to be lengthened in a long sitting posture (similar to that used in Stretch session), even if the *triceps surae* is not fully tensioned. Thus, lower limb fascia could also have played both mechanical and sensory roles on maximal ROM changes.

The main limitation of the present study is the position used to perform the shear-wave measurements (i.e. HIP-neutral). To determine the influence of sciatic nerve stiffness on maximal ankle ROM in dorsiflexion, it would have been more appropriate to measure the sciatic nerve stiffness in the HIP-flexed position that stretches more the sciatic nerve, and therefore contributes to a greater restriction of maximal ROM. However, after considerable piloting, we could not get reliable elastography measurements in this position. Therefore, we performed all the nerve stiffness evaluation in HIP-neutral as previously performed by Andrade *et al*.^4^. Additionally, our results were performed in one site of the sciatic nerve tract, which is further divided into two main branches. As it is known that nerves exhibit localized heterogeneity of tensile properties^34^, the magnitude of the change in nerve stiffness might differ at different locations along the sciatic nerve. Finally, the sciatic ROI used to calculate the nerve shear wave velocity was only adjusted depth-wise during ankle rotation. The same point of the tissue was not followed in a proximal-distal direction. Although this might be considered as a methodological limitation, it has been demonstrated that longitudinal sciatic tract excursion is minimal (about 2 mm) as the ankle is dorsiflexed in HIP-neutral position (e.g. Boyd *et al*.^35^). In addition, in the current study the probe was not removed between pre- to post-intervention nerve stiffness assessments, and thus we are confident that the analyzed region was exactly the same before and after the intervention.

Overall, the results of the present study show that the stretching technique designed to target the sciatic nerve can induce both an acute decrease in the local sciatic nerve stiffness and an increase the maximal ankle ROM in dorsiflexion, without altering the plantar flexors stiffness. Further studies are required to determine whether a chronic nerve stretching protocol (i.e. across weeks) can change tissue stiffness and induce a persistent increase in joint ROM.

## Materials and Methods

### Participants

Fifteen healthy volunteers participated in this study (13 males and 2 females; age: 22 ± 3 years, height: 175 ± 7 cm, weight: 66 ± 7 kg). Participants had no history of significant trauma or surgery to the spine, hip or hamstring region, knee and ankle joints, or symptoms consistent with sciatic or tibial nerve pathology. The straight leg raising and slump tests^36^ had to be negative in all participants, to prevent the inclusion of subjects with pathology. Participants were not engaged in any flexibility training, and were asked to avoid intense exercise 48 h prior to the testing sessions. They were informed about methods used in this study before providing written informed consent. The Institutional Ethics Committee (Tours Ouest I, reference: no. CPP MIP-08) approved the study, and all procedures conformed to the Declaration of Helsinki (last modified in 2013).

### Dynamometer

An isokinetic dynamometer (Con-Trex MJ; CMV AG, Dubendorf, Switzerland) was used to perform passive ankle rotations and to measure the ankle torque. All measurements were performed on the right ankle joint. The lateral malleolus was used to estimate the center of rotation of the ankle, and was aligned with the axis of the dynamometer. The neutral position of the ankle (0°) was defined as an angle of 90° between the footplate and the shank. The foot was firmly strapped such that the potential heel displacement from the dynamometer platform was minimized during the ankle rotations. Ankle angle and torque data were collected at 100 Hz with an analogue/digital converter (ADInstruments, Colorado Springs, CO, USA). A standard goniometer (MSD, Londerzeel, Belgium) was used to set the hip position (flexed at 90° or neutral - i.e. 180°) and knee (fully extended, i.e. 180°) positions. With the exception of the hip joint, the participant’s positioning (i.e. knee and ankle joints) remained unchanged throughout all the testing.

#### Testing position

**Figure 4:**
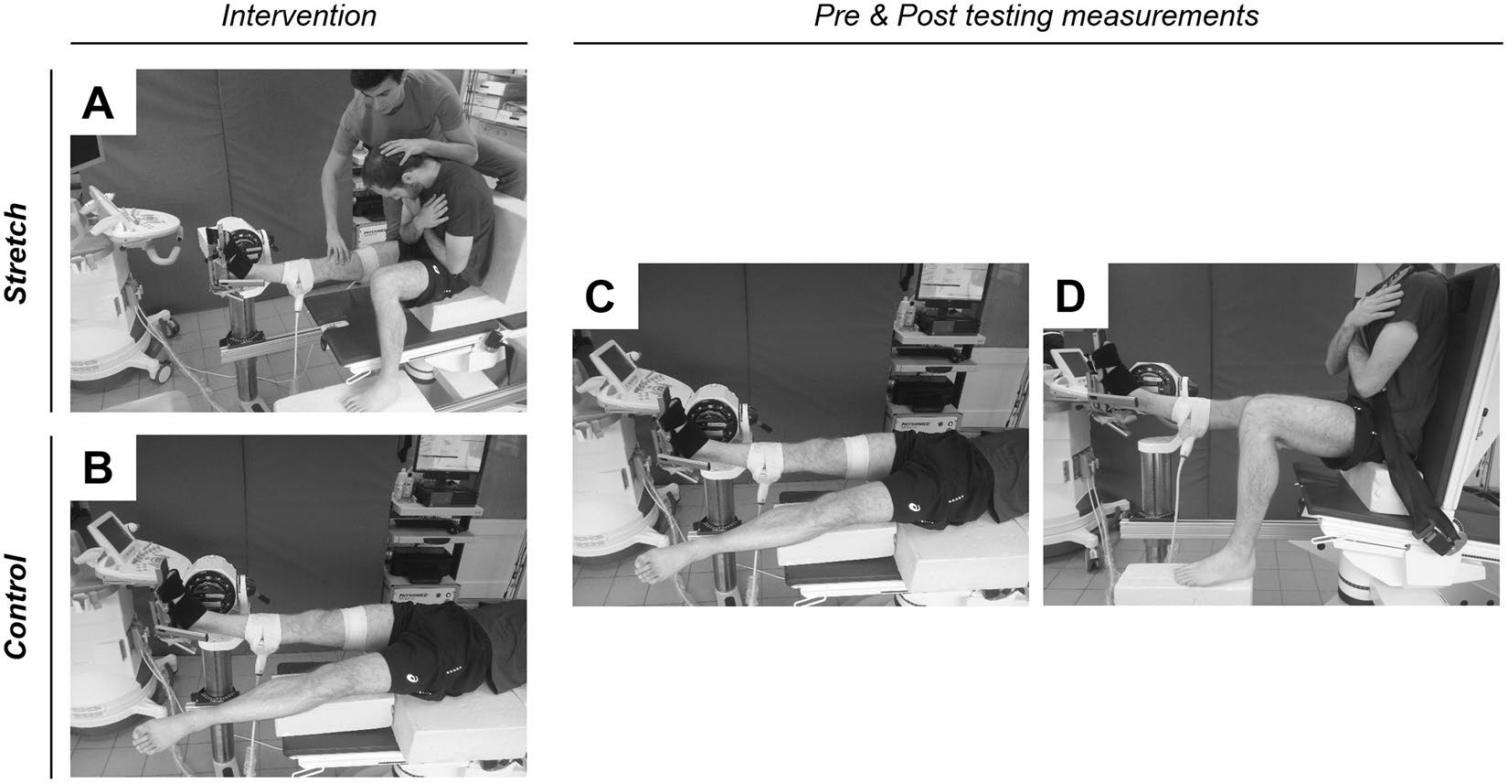
Testing positions used for maximal ankle ROM in dorsiflexion and elastography measurements. (**A**) Stretch position used to stretch the sciatic nerve. Hip and spine were flexed to maximum tolerable stretch limit, and ankle angle was set as neutral; (**B**) HIP-neutral position used as intervention in Control session (ankle was positioned in neutral); (**C**) HIP-neutral position used for maximal ankle ROM, elastography (sciatic nerve, *biceps femoris* and *gasctrocnemius medialis*) and torque measurements before and after the intervention; (**D**) HIP-flexed position used for maximal ankle ROM in dorsiflexion assessment.

Participants were tested for maximal ankle ROM at two hip positions in a random order: HIP-neutral and HIP-flexed. For the HIP-neutral position, participants laid prone (Fig. 4C) on the dynamometer. The contralateral limb remained straight in a relaxed position. The knee was fully extended and strapped securely in this position such that no knee motion occurred during ankle rotations. For the HIP-flexed position (Fig. 1D), participants were seated with the hip flexed at 90° and the knee fully extended. The contralateral limb was flexed to 90° (hip and knee) and remained relaxed. The chest and the waist were strapped to minimize trunk motion during ankle rotations.

### Shear wave elastography

All the assessments were performed in HIP-neutral as previously described^4^ (Fig. 4C). The reason being that sciatic nerve elastography measurements were not reliable in HIP-flexed position (see details in discussion). We assessed the shear wave velocity of the sciatic nerve, the *medial gastrocnemius* (GM) and the long head of *biceps femoris* (BF) muscles. To this end, an Aixplorer ultrasound scanner (version 6.1; Supersonic Imagine, Aix-en-Provence, France) coupled with a linear transducer (4–15 MHz for GM muscle or 2–10 MHz for the sciatic nerve and the BF muscle, Super Linear, Aix-en- Provence, France) was used in shear wave elastography mode. It has been shown that this technique can be used to reliably measure the shear wave velocity of peripheral nerves^4,17,23^. The region of interest (ROI) was first identified in B-mode images. For the sciatic nerve, the transducer was positioned parallel to the nerve fibers on the proximal third of the thigh^4^. The GM was divided in three equal regions calculated from the distance between the distal and the proximal myotendinous junctions, and measurements were performed in the intermediate region as described in by Le Sant, *et al*.^26^. Finally, the shear wave velocity of BF was measured at a location in close proximity to where the sciatic nerve signals were collected. This muscle was examined to determine: (i) if it could be possible to tension the sciatic nerve during ankle dorsiflexion without affecting the muscle’s stiffness; and (ii) whether the nerve stretching intervention could change the nerve stiffness without changing the BF stiffness.

The following shear wave elastography acquisition parameters were set: penetration mode, 100% opacity, no temporal smoothing (persistence = OFF), and intermediate spatial smoothing of 5/9. All the measurements were performed by an experienced ultrasonographer. The transducers were secured to the thigh, for sciatic nerve and BF measurements, and to the lower leg, for GM measurements, with custom-made casts. Strapping was used to hold the ultrasound transducers but care was taken to apply tissue compression as little as possible. Tissue compression was constant throughout each testing session. The transducers were not removed between the measurements, which were therefore performed exactly at the same location before and after the intervention.

An analog trigger signal originating from the ultrasound scanner at each elastography measurement was recorded to ensure a perfect synchronization between shear wave data, ankle angle, ankle torque and surface electromyography (EMG). The maps of the shear modulus were obtained at 1 sample/s and with a spatial resolution of 1 × 1 mm.

### Electromyography (EMG)

To be assured that the lower leg muscles remained inactive during the measurements, myoelectrical activity was assessed using surface EMG. A pair of surface electrodes (Kendall 100 Series Foam Electrodes, Covidien, Massachusetts, USA) was placed over the *gastrocnemius lateralis* (GL), *tibialis anterior* (TA), *soleus* (SOL) and *semitendinosus* (ST) muscles at the location recommended by the Surface EMG for Non-Invasive Assessment of Muscles guidelines^37^. GL and ST were chosen instead of GM and BF because of interference with the locations used for elastography assessments. EMG data were acquired simultaneously with the mechanical data (ADInstruments, Colorado Springs, CO, USA) at a sampling rate of 1 kHz. The EMG signals were amplified (×1000), and band-pass filtered (8–380 Hz).

### Experimental protocol

Each participant performed first one familiarization session. In this session, participants were familiarized with the testing set-up and maximal ankle ROM in dorsiflexion assessments and stretching protocol 24-h prior to their first testing session. Then, participants performed two main experimental testing sessions in a randomized order (established by the toss of a coin) designated ‘Stretch’ and ‘Control’ sessions. These sessions were separated by at least 24-h (mean: 172 hours) and were conducted at the same time of the day. In each testing session, all variables were measured before (pre-testing) and immediately after (post-testing) the stretching or control intervention.

At the beginning of each experimental session, five conditioning ankle rotations at 5°/s were performed in the HIP-neutral position between 40° of plantar flexion and 15° of dorsiflexion^38^. The time between the conditioning rotations and the first measurement of shear wave speed was kept similar between Control and Stretch sessions.

The first session was divided in 4 steps. First, the maximal ROM in dorsiflexion was assessed in both HIP-neutral (Fig. 4C) and HIP-flexed (Fig. 4D) positions (randomized order). Two trials separated by 1-min rest were performed for each testing position. Participants were asked to completely relax with their eyes closed while the ankle was passively rotated at 2°/s from 40° plantar flexion toward maximal dorsiflexion. When the participants reached their onset of any pain response to stretching in the posterior region of the leg^8^, they pressed a button that immediately released the footplate, and hence the associated stretch on the soft tissue structures. Great care was taken to ensure that participants fully understood this procedure during the familiarization session. In both positions, the greatest maximal ankle ROM in dorsiflexion was attained during these trials retained for statistical analysis procedures.

Secondly, over three repetitions, elastography measurements were performed on GM, BF and sciatic nerve (randomized order) while the ankle was passively rotated (2°/s) from 40° of plantar flexion to the maximal ROM in dorsiflexion previously determined in HIP-neutral (see Fig. 4C). Next, participants underwent a total of 6-min (2 × 180 s with a 30 s rest interval) rest (Control session) or nerve stretching (Stretch session) immediately after the pre-testing. Specifically, the nerve stretching was performed by an experienced physiotherapist who passively flexed the hip from the HIP-neutral (i.e. towards a long sitting position), followed by lumbar, thoracic and cervical flexion to the end of range of motion, as dictated by the participant (Fig. 4A). During this manoeuver, the ankle angle was maintained in neutral position (0°) to minimize the stretch of the plantar flexor muscles. This neural stretch technique^5,24^ was performed at the maximal tolerable hip flexion ROM, and used to target the stretch of the sciatic nerve tract with minimal tension within the plantar flexors. The knee of the tested lower limb remained in full extension, while the contralateral lower limb was flexed at 90° throughout the procedure. During the Control session, participants remained relaxed for 6-min in the HIP-neutral position, with the ankle of the tested limb positioned at 0° (Fig. 4B). The contralateral knee was positioned straight in a relaxed position. After 6 minutes, testing identical to the pre-intervention measurements were performed immediately after the Stretch or Control interventions.

Two maximal effort isometric contractions of plantar flexors, and dorsiflexors were performed at the end of each experimental testing session with the ankle in a neutral position with the participants in supine; while knee flexors were tested with the knee fully extended in the same lying position. There was a 1-min rest interval between each maximal effort trial. These MVC contractions were used to normalize the EMG amplitude.

### Data analysis

All data remained coded so that the analyses could be undertaken in a blinded manner. All data were processed using MATLAB scripts (The MathWorks Inc., Natick, USA). Angle and torque signals were low-pass filtered with a second-order Butterworth filter. The root mean square of the EMG signal (RMS EMG) was calculated over 300-ms windows throughout the testing procedures. Artifacts originating from the supersonic push beam (1 sample/s) were discarded from EMG. The RMS EMG values were normalized to that recorded during maximal voluntary isometric contractions.

Shear wave velocity (Vs) was used as an index of muscle and nerve stiffness^12,13^. The Vs is directly linked to the shear modulus (μ)^14^:

1 $${\rm{\mu }}={{\rm{\rho }}\mathrm{Vs}}^{2},$$

where ρ is the estimated density of soft tissues (1000 kg/m^3^). In isotropic homogenous medium, the Young’s modulus (E) can be approximated as three times the shear modulus (μ), as follows

2 $${\rm{E}}=3\,{\rm{\mu }}.$$

However, both muscle and nerve tissues display anisotropic mechanical properties. Thus, the calculation of shear wave velocity and its relationship to the Young modulus can only be considered as an estimate^39,40^. Interestingly, using an *in-vitro* animal preparation, Eby *et al*.^15^ demonstrated that the axial shear modulus of anisotropic skeletal muscle is strongly correlated to the Young’s modulus assessed by traditional materials testing. Similar findings were observed in tendons^41–43^. These results suggest that the shear wave velocity of anisotropic tissues, such as skeletal muscles^15^ or tendons^41–43^, can still provide an accurate estimation of their mechanical properties longitudinally.

**Figure 5:**
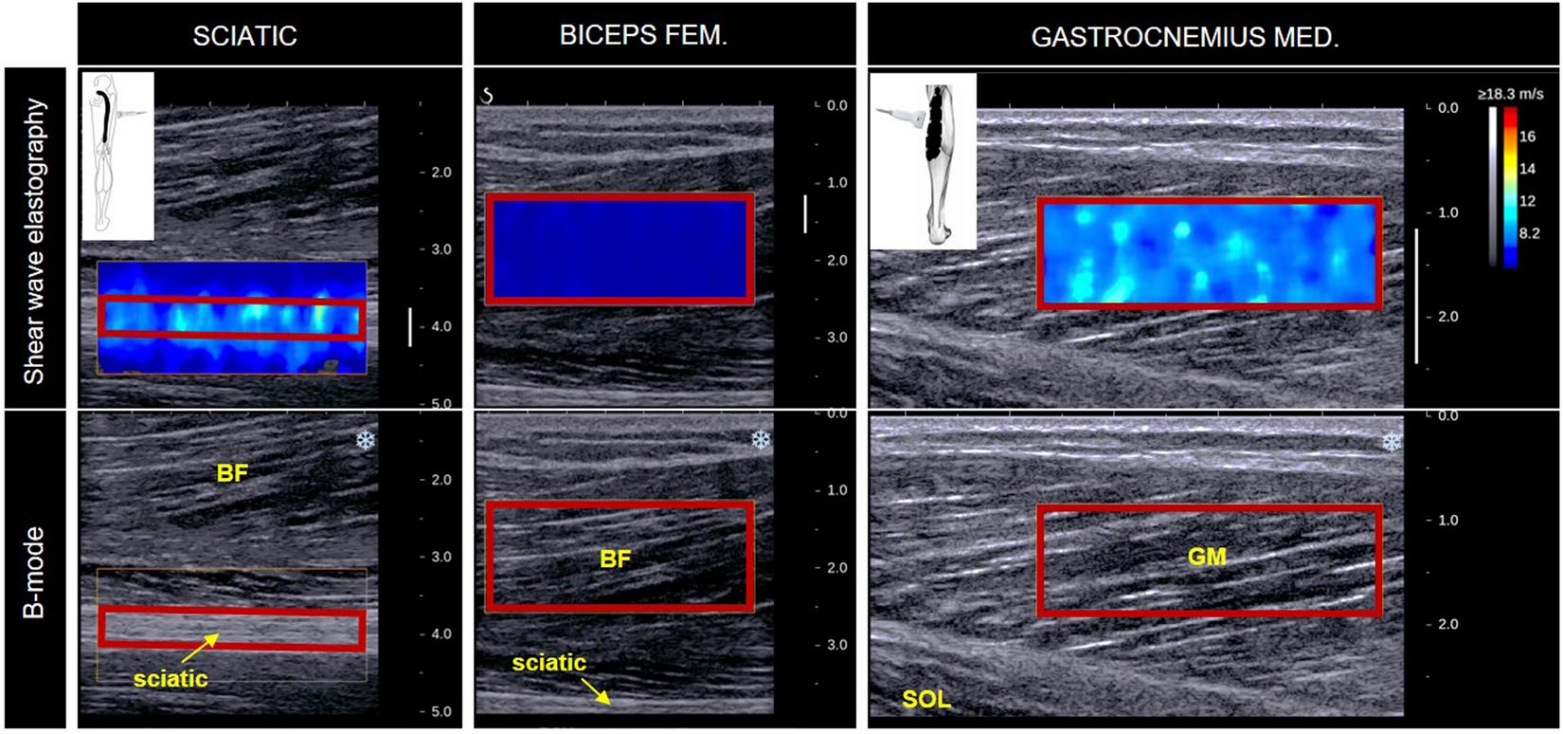
Typical example of sciatic nerve, biceps femoris and gastrocnemius medialis shear wave velocity maps that were collected from participant #8 (at a matched dorsiflexed angle). The region of interest (ROI), corresponding to the rectangles outlined in red, were firstly determined from a B-mode image and subsequently matched in the shear wave elastography image. The biceps femoris shear wave velocity was measured with the probe placed exactly in the same region as the sciatic nerve, by adjusting the ultrasound scan depth-wise. Legend: BF – Biceps femoris; SOL – soleus muscle.

Videos were exported from the Aixplorer’s scanner on ‘mp4’ format, and then sequenced in ‘jpeg’ images. The image processing converted each pixel of the color map into a shear wave velocity value based on the recorded color scale (scale = 0–18.3 m/s). A region of interest (ROI) was first defined on the first map as the largest muscle or nerve area (Fig. 5). Then this ROI was manually tracked every second during the recording performed for each ankle rotation in dorsiflexion. Each ROI was then inspected for artifacts (saturation or void areas). If artifacts were present in any of the images to be analyzed within a recording, the ROI was reduced in size to exclude the area of artifact from all images within that recording^4^. The shear wave velocity data extracted from this ROI were averaged to obtain a representative value.

When the shear wave length (related to the stiffness) is much larger than the mean tissue thickness, as it is the case for the tendon, the wave propagation is guided along the tendon and its velocity decreases due to successive reflections at the tendon boundaries^44,45^. Within this context, the relationship between group velocity of the shear wave and tendon elasticity is affected by tendon thickness; and thus equation 1 cannot be true. Importantly, the nature of the relationship between thickness, elastic modulus and shear wave velocity is unknown. Even though guided waves have been well described for tendons^44^, to the best of our knowledge, they have not been described for nerves. Because the sciatic nerve is less stiff than the tendon^4,45,46^, we believe that shear waves are guided less in nerves than the tendons. Therefore, our results are reported as shear wave velocity^4^. In addition, the mean sciatic nerve thickness was calculated using the B-mode ultrasound images to ensure that pre- to post-intervention changes in shear wave velocity would not have been influenced by nerve thickness adaptations to acute stretching. Specifically, the nerve thickness was calculated from the distance between sciatic nerve boundaries on the locations of shear wave velocity measurements.

The sciatic nerve, GM and BF shear wave velocity, ankle torque and RMS EMG were calculated every 2°, from 40° of plantar flexion to the maximal ankle ROM in dorsiflexion. For each participant, the maximal ankle ROM in dorsiflexion attained in pre-testing (Control or Stretch sessions) was used to examine the pre- to post-effects of the interventions. This corresponds to 100% of the pre-testing ankle ROM in dorsiflexion (i.e. dotted gray lines in Fig. 1). At this common dorsiflexion angle between pre- and post-testing, sciatic nerve, GM and BF shear wave velocity, ankle torque and RMS EMG values were determined and then used for statistical analysis purposes.

### Statistics

The IBM SPSS software (version 20.0; IBM Corporation, New York, USA) was used for the statistics procedures. Distributions consistently passed the Shapiro-Wilk normality test. All data are thus reported as mean ± standard deviation (SD).

Two-way ANOVA with repeated measures [session (Stretch, Control) × time (pre, post)] was performed to identify changes in maximal ROM in dorsiflexion after the intervention in both testing positions (HIP-neutral and HIP-flexed). Nine two-way ANOVAs with repeated measures [session (Stretch, Control) × time (pre, post)] were performed to examine the effect of the intervention on the sciatic nerve and muscles shear wave velocity (GM, BF), ankle torque, sciatic nerve thickness, and RMS-EMG of GL, SOL, TA and ST. These analyses were performed at the same ankle angle, i.e. 100% of the pre-testing ankle ROM in dorsiflexion. The partial eta square (_p_η^2^) values were reported as a measure of the effect size of the ANOVA’s findings. Small, medium and large effects were considered for _p_η^2^ = 0.01, _p_η^2^ = 0.06 and _p_η^2^ ≥ 0.14, respectively^47^. Post hoc analyses subsequent to the ANOVAs were performed when appropriate using the Bonferroni correction for multiple comparisons. Cohen’s d were calculated to determine the effect size for Bonferroni post hoc paired comparisons.

In addition, Pearson’s correlation was used to test the correlation between the relative decrease (in %) in sciatic nerve shear wave velocity observed after the nerve stretch (Stretch session) and the relative increase (in %) in maximal ROM in dorsiflexion assessed in both HIP-flexed and HIP-neutral positions. Similar correlations were tested for Control session. The statistical significance was set at P < 0.05.

The datasets generated during and/or analysed during the current study are available from the corresponding author on reasonable request.
